# Supplementary material for: From sequence to enzyme mechanism using multi-label machine learning
Source: BMC Bioinformatics. 2014 May 19;15:150. doi: 10.1186/1471-2105-15-150 (PMC4229970; doi:10.1186/1471-2105-15-150)
Supplement: Additional file 2 — Java code of ml2db. Additional file ml2db_code.tar.gz contains the Java source code to run the multi-label machine learning experiments and save the results to database. The code’s Javadoc is included. [file 1471-2105-15-150-S2.zip › additional file 2/ml2db/ml2db/doc/main/ml2db/mulan/mulanarff/package-summary.html]

main.ml2db.mulan.mulanarff


JavaScript is disabled on your browser.


- Overview
- Package
- Class
- Use
- Tree
- Deprecated
- Index
- Help

- Prev Package
- Next Package

- Frames
- No Frames

- All Classes

# Package main.ml2db.mulan.mulanarff

- Class Summary

  | Class | Description |
  | --- | --- |
  |  |  |
  | --- | --- |
  | AttributeUtils |  |
  | IdInstance | A Weka instance with identifier. |
  | IdMultiLabelInstances | A set of identified Mulan Multi Label instances (each instance has a unique string identifier). |
  | ImportMulanArff |  |
  | InstanceUtils | Class |
  | MulanArff | Generates an Attribute Relation Format File (ARFF) in Mulan format (multiple class labels per instance). |
  | MulanArffProperties | Class |
  | MulanAttributeFactory | In the Mulan ARFF, the class attribute is not generated. |
  | MulanDataSetDbLoader | Loads data from database to a Mulan data factory |
  | MulanDataSetGenerator | Class |
  | MulanDataSetManager | This class generates a data set (instances, attributes, classes) in Mulan style (for multi-label machine learning). |
  | MulanInstancesFiller | Class |
  | NeighboursPrinter | Class to store the neighbours of an instance and print them in a human/graphviz readable format. |
  | NeighboursPrinterTest | Class |
  | Statistics | Class exteded from Mulan  Class for calculating statistics of a multilabel dataset |

- Overview
- Package
- Class
- Use
- Tree
- Deprecated
- Index
- Help

- Prev Package
- Next Package

- Frames
- No Frames

- All Classes
